# Lessons From Pediatric MDS: Approaches to Germline Predisposition to Hematologic Malignancies

**DOI:** 10.3389/fonc.2022.813149

**Published:** 2022-03-09

**Authors:** Serine Avagyan, Akiko Shimamura

**Affiliations:** Dana-Farber/Boston Children’s Hospital Cancer and Blood Disorders Center, Harvard Medical School, Boston, MA, United States

**Keywords:** germline, inherited bone marrow failure syndromes, predisposition syndromes, myelodysplastic syndrome, hematologic malignancy

## Abstract

Pediatric myelodysplastic syndromes (MDS) often raise concern for an underlying germline predisposition to hematologic malignancies, referred to as germline predisposition herein. With the availability of genetic testing, it is now clear that syndromic features may be lacking in patients with germline predisposition. Many genetic lesions underlying germline predisposition may also be mutated somatically in *de novo* MDS and leukemias, making it critical to distinguish their germline origin. The verification of a suspected germline predisposition informs therapeutic considerations, guides monitoring pre- and post-treatment, and allows for family counseling. Presentation of MDS due to germline predisposition is not limited to children and spans a wide age range. In fact, the risk of MDS may increase with age in many germline predisposition conditions and can present in adults who lack classical stigmata in their childhood. Furthermore, germline predisposition associated with *DDX41* mutations presents with older adult-onset MDS. Although a higher proportion of pediatric patients with MDS will have a germline predisposition, the greater number of MDS diagnoses in adult patients may result in a larger overall number of those with an underlying germline predisposition. In this review, we present a framework for the evaluation of germline predisposition to MDS across all ages. We discuss characteristics of personal and family history, clinical exam and laboratory findings, and integration of genetic sequencing results to assist in the diagnostic evaluation. We address the implications of a diagnosis of germline predisposition for the individual, for their care after MDS therapy, and for family members. Studies on MDS with germline predisposition have provided unique insights into the pathogenesis of hematologic malignancies and mechanisms of somatic genetic rescue *vs.* disease progression. Increasing recognition in adult patients will inform medical management and may provide potential opportunities for the prevention or interception of malignancy.

## Introduction

Although historically considered in the context of pediatric myelodysplastic syndromes (MDS), genetic predisposition to hematologic malignancies, including MDS, is now recognized to manifest across the age spectrum (1–4). Germline etiology has been associated with up to 15% of MDS diagnoses (1, 2, 5, 6), a number that undoubtedly will increase with the availability of clinical genetic testing and our understanding of MDS pathobiology. Most germline MDS predisposition conditions were originally characterized in patients with severe early-onset presentations with specific syndromic features. However, the absence of classic physical features or family history of hematologic malignancies does not preclude consideration of germline predisposition, due to clinically cryptic phenotypes or *de novo* germline mutations.

MDS associated with germline predisposition is now classified separately in the 2016 World Health Organization system (7). Recognition of an underlying germline predisposition has broad implications for both patients and their families. As a hematopoietic stem cell transplantation is the curative therapy for many patients with MDS, donor choices will be influenced by the underlying genetics, which may affect other family members. Lastly, the diagnosis of germline MDS predisposition guides family counseling and family planning and allows appropriate monitoring of affected asymptomatic individuals to maintain health. We present here a framework for evaluating MDS that is applicable to both pediatric and adult patients.

## Predisposition to MDS and Other Hematologic Malignancies Is not Restricted to Pediatric Patients

Consider the following cases presenting to an adult clinic: (1) a 27-year-old sibling of a 19-year-old man with MDS is undergoing evaluation as a possible donor for a stem cell transplantation. This sibling is unexpectedly found to have macrocytic anemia and thrombocytopenia. A subsequent bone marrow evaluation reveals multilineage dysplasia with trisomy 8 consistent with MDS; (2) A 57-year-old woman with MDS reports that her 16-year-old grandson was treated for acute myeloid leukemia. Should these cases raise concern for predisposition syndromes? The first case of both a proband and sibling with MDS would be suspicious for an underlying genetic etiology, which, in this case, was a germline *GATA2* mutation. The second case of multiple family members with myeloid malignancy should also raise consideration of germline predisposition, despite the adult-onset MDS diagnosis. This family harbored a germline mutation in *RUNX1* that manifested across generations.

Approximately 90% of MDS is diagnosed in adults over the age of 60 years, with only 6% diagnosed in those younger than 50 years (8). It is increasingly recognized that patients with conditions previously considered pediatric diseases may first come to medical attention with adult-onset MDS. The diagnosis of MDS may be the first presentation of the underlying germline predisposition or may be the hematologic presentation of a syndrome unrecognized with subtle or non-hematologic manifestations, like short-stature in Shwachman–Diamond syndrome (SDS) (2) or lymphedema in *GATA2* deficiency (9). Furthermore, due to high degree of variability of penetrance of a specific phenotype, manifestations of the different aspects of the syndrome may present at different ages. Even within single families, the genotype often poorly correlates with the phenotype in germline predisposition. For example, *RUNX1*-familial platelet disorder (*RUNX1*-FPD), a syndrome associated with germline mutations in *RUNX1*, may present with MDS, AML, T-cell acute lymphoblastic leukemia, or other lymphoid cancers between the ages of 9 and 65 (10). In telomere biology disorders (TBD), MDS and myeloid leukemia were the most commonly diagnosed cancers, with a median age at diagnosis of 53 years (range of 12–71 years old) (11). *TERT* mutations were identified in 2.7% of adult patients with MDS (12). *DDX41* mutations associated with MDS and AML are characterized by presentation in older adulthood, often without any family history (13, 14). Identification of germline mutations in *DDX41* in familial malignancies presenting at older ages has shifted the paradigm of germline predisposition affecting only children and young adults. Over 70 families with germline *DDX41* mutations have been reported, with a median age of MDS onset of 65 years (range of 41–88 years old), similar to the median age of onset of sporadic MDS in early 70s (15, 16). Thus, predisposition syndromes should be considered in adult patients with MDS to inform medical management.

## A Diagnostic Framework to Evaluate a Suspected Germline Predisposition to MDS and Other Hematologic Malignancies

MDS may be the first presenting clinical feature of a germline predisposition condition. The diagnostic evaluation for a potential genetic predisposition condition integrates the history, laboratory tests, histopathological findings in the bone marrow, and genetic testing. Focusing on the syndromes that span pediatric and adult hematology, we will focus on clues to the diagnosis of germline predisposition. Since many of these syndromes have been described previously, we will not review them in detail here but refer the reader to these excellent references (17–19).

### Clues in the Clinical Presentation

In general, the presenting signs and symptoms of MDS do not differ in patients with germline predisposition and include nonspecific clinical findings of fatigue, pallor, malaise, fevers, bleeding, increased bruising, weight loss, and anorexia. The marrow is often hypocellular in pediatric MDS or MDS in germline predisposition, in contrast to adult-onset MDS, which is more commonly hypercellular. Adult patients with hypocellular MDS have significantly more pronounced cytopenias at presentation compared to those presenting with normocellular or hypercellular marrows (20). It is yet unknown whether the small subset of hypocellular MDS in adults, between 10% and 20% of all MDS (21, 22), are more likely to be related to germline predisposition. For example, germline *DDX41-*related adult-onset MDS is associated with a hypocellular marrow (14, 16). The duration of specific clinical symptoms may allow insight into the chronicity of cytopenias. In patients with history of neutropenia in childhood and adolescence, information on the length and dose of granulocyte-colony stimulating factor (G-CSF) usage is helpful in the evaluation of a patient with new MDS, as it may suggest germline mutations associated with severe congenital neutropenia (SCN) due to germline *ELANE* mutations. Prolonged use, particularly with high doses, of G-CSF in SCN is associated with a 22% cumulative incidence of MDS or secondary AML after 15 years of therapy (23). The risk of MDS development is associated with a somatic acquisition of mutations in *CSF3R*, the receptor for G-CSF (24–26). A history of prior cytopenias or unexplained macrocytosis may also serve as a clinical clue for germline predisposition. Elevated hemoglobin F may be associated with inherited germline predisposition (27) but can also be elevated in MDS and, thus, should be interpreted with caution (28–30).

Past medical history of bleeding, easy bruising, or diagnosis of “chronic immune thrombocytopenia” is a feature associated with mutations in transcription factors important for megakaryopoiesis and platelet function, including *RUNX1, ANKRD26*, and *ETV6* (31–33). Patients with germline mutations in these genes may have quantitative and/or qualitative platelet defects, manifesting with easy bruising, heavy menses in women, or other bleeding symptoms, which may be out of proportion to the platelet count. Amegakaryocytic thrombocytopenia, often with radioulnar synostosis, is associated with germline *MECOM* mutations, presenting as congenital bone marrow failure in early childhood (34, 35). In rare case reports, mutations in *MECOM* have been described in association with adult-onset MDS (36). A history of thrombosis has been reported in *GATA2* deficiency, related to cerebrovascular accidents, deep vein thrombosis, pulmonary embolism, and portal vein thrombosis (9). Clinical features of *GATA2* deficiency may include recurrent infections, atypical mycobacterial infections, warts, pulmonary alveolar proteinosis, or lymphedema. Although not with clear association to vascular dysfunction or immune deficiencies, premature labor and miscarriages have been described in *GATA2* deficiency (9, 37) and other germline conditions (38–40).

### Personal History of Cancer

Past medical history of malignancy should be investigated as part of the evaluation of germline predisposition (3). In *RUNX1-*FPD, cases of secondary malignancy with MDS after therapy for acute lymphoblastic leukemia (ALL) have been described, particularly in survivors of childhood T-cell ALL (10, 41, 42). A history of excessive toxicities with chemotherapy or radiation may also suggest a genetic predisposition such as Fanconi anemia (FA). A history of multiple malignancies should raise concern for possible germline predisposition. Solid organ cancers are associated with conditions such as FA [squamous cell carcinomas of head and neck, gastrointestinal (GI) and genital tract, and other solid organ cancers], TBD (head/neck and GI) (43), and Diamond–Blackfan anemia (DBA), particularly in adults (44). In the case of FA, patients may have subclinical mild cytopenias or normal blood counts in childhood, only to present with a solid organ cancer in adulthood, and experience severe protracted pancytopenia with chemotherapy or radiation (45). The diagnosis of a germline predisposition in a patient with a past medical history of malignancy guides subsequent cancer monitoring. This is particularly important in syndromes associated with mutations affecting genomic instability such as FA and Li–Fraumeni syndrome (LFS).

### Giveaways From the Past: Thinking Outside of the Hematopoietic System

A detailed investigation of the past medical history of a patient with MDS may also identify non-hematologic clinical manifestations of germline predisposition (**Table 1**).

**Table 1:** Clinical features of and genetic mutations underlying germline conditions with predisposition to myeloid neoplasms.

Conditions with germline mutations associated with predisposition to MDS^a^	Gene(s)	Inheritance	Hematologic malignancies reported	Pre-leukemia hematopoietic features	Immunologic features	Non-hematologic clinical features (may be entirely absent)	Somatic genetic rescue/ reversion
**Germline predisposition conditions associated with both childhood and adult onset myeloid neoplasms**							
Fanconi anemia	*FANCA*, *FANCB*, *FANCC, FANCD1/BRCA2, FANCD2*, *FANCE*, *FANCF*, *FANCG*, *FANCI*, *FANCJ/BRIP1, FANCL*, *FANCM, FANCN/PALB2, FANCO/RAD51C, FANCP/SLX4, FANCQ/ERCC4, FANCR/RAD51, FANCS/BRCA1, FANCT/UBE2T, FANCU/XRCC2, FANCV/REV7, FANCW/RFWD3, FANCY/FAP100*	AR AD (*FANCR/RAD51)* X-linked recessive (*FANCB*)	MDS, AML, T-ALL	Cytopenia(s), bone marrow failure	CVID, abnormal lymphopoiesis (46)	Short stature, microcephaly, CNS structural abnormalities, developmental delay, microphthalmia and other eye abnormalities, hearing loss, abnormal facies, cardiac structural abnormalities, GI abnormalities including intestinal malrotation, GU abnormalities, upper limb abnormalities including absent or abnormal thumb, polydactyly, hypoplastic thenar eminence, lower limb abnormalities, café-au-lait macules and other skin hyper- or hypopigmentation, squamous cell carcinomas of head and neck, GI and genital tract, hepatic tumors, and other cancers	Reported (47–49)
Diamond-Blackfan anemia	*GATA1* (50), *RPL5*, *RPL11*, *RPL15*, *RPL18*, *RPL26*, *RPL27*, *RPL31*, *RPL35*, *RPL35A* *RPS7*, *RPS10*, *RPS15A*, *RPS17*, *RPS19*, *RPS24*, *RPS26*, *RPS27*, *RPS28*, *RPS29*, *TSR2* (51)*,^b^*	AD X-linked (*TSR2)*	MDS, AML, HL, NHL, ALL	Macrocytic anemia, reticulocytopenia, elevated fetal hemoglobin (HgF), elevated erythrocyte adenosine deaminase activity	Hypogammaglobulinemia, lymphopenia (52)^c^	Short stature, failure to thrive, abnormal facies, abnormal thumb (normal radius), limb abnormalities, cardiac structural abnormalities, GU abnormalities, solid organ cancers (particularly soft tissue sarcomas)	Reported (53–55)^d^
Shwachman-Diamond and related syndromes	*SBDS*, *DNAJC21*, *EFL1*, *SRP54*	AR AD (*SRP54)*	MDS, AML (currently no reports in *EFL1* or *SRP54)*	Neutropenia, other cytopenias, bone marrow failure	Immune disorders (56), immunodeficiency (57)	Short stature, exocrine pancreatic insufficiency, skeletal dysplasias, thoracic dystrophy, osteopenia, transaminitis/hepatomegaly early in life, eczema, variable neurocognitive delays, inflammatory complications	Reported (58)^e^
Telomere biology disorders	*ACD/TPP1*, *CTC1*, *DKC1*, *NAF1*, *NHP2/NOLA2*, *NOP10/NOLA3*, *PARN*, *RPA1* (59), *RTEL1*, *STN1* *TERC*, *TERT*, *TINF2*, *WRAP53/TCAB1*, *ZCCHC8*	AD (*TERT, TERC, TINF2, RTEL1, ACD, PARN, NAF1, STN1)*, AR (*TERT, NOP10, NHP2, WRAP53, CTC1, RTEL1, ACD, PARN)*, X-linked recessive (*DKC1)*	MDS, AML	Bone marrow failure, aplastic anemia, macrocytosis, cytopenias	Lymphopenia, B-cell lymphopenia, hypogammaglobulinemia, and decreased T-cell function	Nail dysplasia, early greying, abnormal skin pigmentation, oral leukoplakia, GI dysfunction, neuropsychiatric disorders, interstitial lung disease (adult presentation), emphysema (adult presentation), cryptogenic cirrhosis (adult presentation), Hoyeraal-Hreidarsson Syndrome (cerebellar hypoplasia in young children), Revesz syndrome (bilateral exudative retinopathy in young children, typically associated with *TINF2)*	Reported (59–62)
*GATA2* deficiency syndrome	*GATA2*	AD	MDS, AML, CML, CMML, JMML, B-ALL	Monocytopenia, dendritic cell, B-cell and natural killer cell deficiency, chronic neutropenia, bone marrow failure	Recurrent bacterial, fungal, and viral infections associated with immunodeficiencies; Mycobacterial infections, autoimmune features including thyroid issues, rheumatological features with Lupus-like arthritis, panniculitis (63)	Lymphedema; sensorineural deafness; urogenital tract anomalies (e.g. hydrocele); pulmonary alveolar proteinosis; behavioral issues, neurologic issues, thrombosis, premature labor and miscarriage	Reported (64)
Severe congenital neutropenia	*ELANE*, *G6PC3* *GFI1*, *HAX1*, *SRP54*, *CSF3R^f^*	AD (*ELANE, GFI1, SRP54*) AR (*HAX1, G6PC3, CSF3R)*	MDS, AML^g^ (no reports in *SRP54*, see below for germline *CSF3R)*	Neutropenia	Lymphopenia and immunodeficiency (*GIF1)*	osteopenia (*ELANE)*, seizures and other neurologic disorders (*HAX1*), exocrine pancreatic insufficiency (*SRP54)*, congenital cardiac malformations and GU abnormalities (*G6PC3)*, Dursun syndrome (familial primary pulmonary hypertension, leucopenia, and atrial septal defect, *G6PC3)*	None reported
*RUNX1*-familial platelet disorder	*RUNX1*	AD	MDS, AML, T-ALL, T-NHL, CLL, HL, B-ALL	Thrombocytopenia, platelet function defect	Eczema, allergies, psoriasis, autoimmune disorders (65)		Reported (66)
Thrombocytopenia 2	*ANKRD26*	AD	MDS, AML, CML, CLL, CMML	Thrombocytopenia, mild bleeding			None reported
Thrombocytopenia 5	*ETV6*	AD	B-ALL, MDS, AML, CMML, PV	Thrombocytopenia, bleeding		Rare case reports of solid organ cancers	None reported
*DDX41*-associated familial MDS/AML	*DDX41*	AD	MDS, AML, CMML, lymphoma, MM				None reported
Li-Fraumeni syndrome	*TP53*	AD	MDS (often therapy-related), AML (often therapy-related), B-ALL (hypodiploid), T-ALL (early precursor), lymphoma			Solid organ cancer	None reported
ERCC6L2	*ERCC6L2*	AR	MDS, AML (particularly AEL^h^)				None reported
Xeroderma pigmentosum	*XPC^i^*	AR	MDS, AML, ALL (67, 68), B cell lymphoma (68)			Photosensitive skin, high frequency of skin tumors, microcephaly, diminished or absent deep tendon reflexes, progressive sensorineural hearing loss, progressive cognitive impairment	None reported
Bloom syndrome	*BLM*	AR	MDS, AML, lymphoma, ALL		Immunodeficiency	Short stature, microcephaly, telangiectatic rash over face, nose, arms, sun-sensitive rash, pulmonary disease, high-pitched voice, hypogonadism	None reported
CEBPA	*CEBPA*	AD	AML				None reported
**Germline predisposition syndromes typically associated with childhood myeloid neoplasms**							
*SAMD9* syndrome	*SAMD9*	AD	MDS, AML	Bone marrow failure, pancytopenia, clonal hematopoiesis with monosomy 7 or acquired revertant mutations in SAMD9	Immunodeficiency	Restriction of growth, adrenal hypoplasia, genital abnormalities, enteropathy	Reported (4, 5, 69)
*SAMD9L* syndrome^k^	*SAMD9L^l^*	AD	MDS, AML	Bone marrow failure, pancytopenia, clonal hematopoiesis with monosomy 7 or acquired revertant mutations in SAMD9L	B-cell immunodeficiency (typically associated with familial ataxia pancytopenia (ATXPC) syndrome related to *SAMD9L*)(70), autoinflammatory disease (71)	Cerebral calcifications (typically associated with familial ataxia pancytopenia (ATXPC) syndrome related to *SAMD9L*)(70)	Reported (4, 72, 73)
*MECOM*-associated syndrome	*MECOM*	AD	MDS	Congenital bone marrow failure, amegakaryocytic thrombocytopenia	Immunodeficiency (34)	Limb dysmorphisms, including radioulnar synostosis, hearing impairment, cardiac abnormalities	None reported
Neurofibromatosis 1	*NF1*, *SPRED1*	AD	MDS, JMML			Optic gliomas, CNS tumors, malignant peripheral nerve sheath tumors, other solid tumors, café-au-lait spots, axillary and inguinal freckling, neurofibromas, Lisch nodules, bony dysplasia	None reported
X-linked neutropenia	*WAS*	X-linked	MDS, AML	Monocytopenia, platelet function defect with bleeding	Immunodeficiency, T-cell lymphopenia and decreased function		None reported
Noonan syndrome	*BRAF*, *KRAS*, *MAP2K1*, *NRAS*, *PTPN11*, *RAF1*, *SOS1*	AD	MDS, JMML, AML, MPN			Facial dysmorphism, short stature, cardiac, broad neck, thoracic, cryptorchidism, Noonan-like coagulopathy, solid organ tumors	None reported
Noonan-like	*CBL* *SHOC2*	AD	JMML (74)			impaired growth, developmental delay, cryptorchidism (*CBL)*(74), hair anomalies and skin pigmentation (*SHOC2*)(75)	None reported
Constitutional mismatch repair deficiency	*MLH1*, *MSH2*, *MSH6*, *EPCAM* *PMS2*	AR	AML, ALL, lymphoma (76)		Decreased IgG and IgA (77)	Gastrointestinal (colon), ovarian, uterine, CNS tumors and other solid organ cancers, café-au-lait macules, skin hyper- and hypopigmentation, pilomatricomas, agenesis of the corpus callosum	
**Reported germline mutations with potential for predisposition to myeloid neoplasms with currently limited data**							
Congenital amegakaryocytic thrombocytopenia	*MPL*	AR	MDS (78)	Thrombocytopenia, bone marrow failure		brain, ocular and orbital anomalies (strabismus, nystagmus), facial abnormalities (78)	None reported
Thrombocytopenia absent radii	compound (bi-allelic) inheritance of one of two noncoding single-nucleotide variants (due to proximal 1q21.1 deletion) and a null allele in *RBM8A*	AR	MDS (79), AML (80), ALL (81)	Thrombocytopenia	Cow's milk intolerance	Absent radii, preservation of the thumb, other birth defects (hypoplastic ulnae, hypoplastic humeri, phocomelia, abnormal shoulders, bowed legs, hip dysplasias, abnormal knees), abnormal facies, renal malformations, gastroenteritis,	None reported
TET2	*TET2*	AD	AML, CMML, T-cell lymphoma (82)		Immunodeficiency (children)(83)		None reported
*SRP72*-associated familial aplasia and myelodysplasia	*SPR72*	AD	MDS, AML (84)	Bone marrow failure, aplastic anemia		Sensorineural hearing loss	None reported
CSF3R	*CSF3R^f^*	AD	MDS, MM, ALL (85)				None reported
Tatton-Brown-Rahman syndrome	*DNMT3A*	AD	AML	Non-anemic macrocytosis, mild lymphocytopenia, mild neutrophilia (single cohort)(86)		Overgrowth, developmental disorders, neuropsychiatric disorders	Reported (87)
*MYSM1* deficiency	*MYSM1*	AR	Myelodysplastic features in marrow (88)	Bone marrow failure	Immunodeficiency, particularly severe B-cell lymphopenia and reduced marrow B-cell progenitors	midface hypoplasia, low-set ears, hypoplasia of the orbital floor, short neck, reduced cerebral volume on MRI, bony abnormalities, osteopenia, deep-set thumb, accessory papilla of the breast, gingiva hyperplasia, neurodevelopmental delay	Reported (89)
*MBD4* deficiency	*MBD4*	AR	AML with myelodysplastic features (90)	Clonal hematopoiesis			None reported

a Data on individual gene associations with MDS are limited. Please note that the table does not provide a comprehensive list of all clinical features and the reader should referred to other excellent reviews.

b Gain-of-function mutations in TP53 have been reported in two patients with pure red cell aplasia, growth retardation and other clinical features, however, there is limited information to classify TP53 as DBA-associated gene.

c Clinically significant infections seen in 3 patients of 107 total with large chromosomal deletions, encompassing RPL35A in 2 cases, and RPS19 in 1 case.

d A case report describes a somatic reversion event in a chromosomal region involving RPL4 in a patient with possible clinical features of DBA, although only a single timepoint of normocytic mild anemia and no further information on hematologic parameters (55).

e The del20q, a common chromosomal loss described in SDS which encompasses the EIF6 gene, has not been shown to cause functional reversion (58, 92–96), except in a single case series of 6 patients with mild effect on anemia (58). Loss of EIF6 does not confer clinically significant revertant phenotype, however, EIF6 mutant clones gain competitive fitness in SDS patients (97, 98).

f Biallelic hypomorphic mutations in CSF3R are associated with severe congenital neutropenia where no cases of MDS or AML have been reported. Heterozygous CSF3R germline mutations have been associated with single kindred with MDS, MM and ALL (85).

g Increased risk of MDS and AML has been reported in patients with severe congenital neutropenia who are on >8mcg/kg/day G-CSF therapy for a prolonged periods of time (23–26). However, cases before G-CSF use have also been reported.

h Acute erythroid leukemia is associated with a founder mutation in ERCC6L2, c.1457delT (p.Ile486ThrfsTer36), in a cohort from Finland (99).

i There are other genes associated with xeroderma pigmentosum but their association with myeloid neoplasms is not clear.

j Also previously known as MIRAGE syndrome (Myelodysplasia, Infection, Restriction of growth, Adrenal hypoplasia, Genital problems, and Enteropathy)

k Also previously known as the Familial myelodysplastic syndrome/monosomy 7

l SAMD9L has now been recognized to underlie three distinct syndromes which are familial ataxia pancytopenia (ATXPC) syndrome with clinical features of B-cell immunodeficiency and cerebral calcifications (70), familial myelodysplastic syndrome/monosomy 7 without neurological phenotype (72), and the SAMD9L-associated autoinflammatory disease (71).

AD, autosomal dominant; AR, autosomal recessive; ALL, acute lymphoblastic leukemia; AEL, acute erythroid leukemia; AML, acute myeloid leukemia; HL, Hodgkin lymphoma; NHL, non-Hodgkin lymphoma; MDS, myelodysplastic syndromes; CNS, central nervous system; GI, gastrointestinal; GU, genitourinary; CLL, chronic lymphocytic leukemia; CMML, chronic mono-myelocytic leukemia; PV, polycythemia vera; MM, multiple myeloma; MPN, myeloproliferative neoplasms.

*Growth and Congenital Anomalies*. Many germline predisposition conditions are associated with a history of intrauterine growth retardation, failure to thrive in childhood, and short stature. Congenital malformations, including skeletal anomalies, abnormal digits, cardiac structural defects, and gastrointestinal (GI) or genitourinary (GU) abnormalities, are important to recognize, many of which may have been repaired in childhood. Such malformations may be part of early presentations of FA (99), TBD (100), DBA (101, 102), and other germline predisposition (**Table 1**). A history of hydrocele in infancy in patients with *GATA2* deficiency may be one of the earliest manifestations of the lymphatic dysfunction of that syndrome that is often unrecognized.

*Immunologic Features*. Many predisposition conditions are associated with immunodeficiency, which may manifest with recurrent infections or immune dysregulation, or may be clinically asymptomatic but detected by laboratory testing for lymphocyte counts, lymphocyte subsets (T-, B-, and NK cells), and immunoglobulin levels. *GATA2* deficiency syndrome, for instance, is associated with atypical mycobacterial infections and warts (genital and extragenital) due to the decreased natural killer and dendritic cells, as well as B-cell lymphopenia (9, 103). Immunodeficiency has also been described in TBD (104–107) and in germline *TET2* deficiency (83), particularly in childhood for the latter. There may also be autoimmune or inflammatory manifestations of germline predisposition conditions that are actively being studied. These include thyroid disease [*GATA2* deficiency (9)], eczema and allergies [*RUNX1-*FPD (10, 65)], psoriasis [*RUNX1-*FPD (10)], panniculitis, and lupus-like arthritis [*GATA2* deficiency (103, 108)], and others listed in **Table 1**. Laboratory screening of immunological abnormalities is recommended below and should be considered in the workup of MDS patients.

*Beyond the Blood*. Given the multifaceted functional roles of the underlying genes in germline predisposition, it is not surprising to find tissues other than blood being affected in these conditions. **Table 1** lists examples of non-hematologic manifestations of these conditions. A history of extremity edema, typically unilateral and sometimes diagnosed as hemihypertrophy, or genital edema (for example, hydrocele) may be related to germline *GATA2* deficiency-associated lymphedema (9, 63). *GATA2* is expressed in the lymphatic system (109, 110) and is thought to underlie various forms of lymphatic dysfunction, occasionally with atypical presentations such as swelling on the face or preauricular region (111). Adult-onset non-hematologic manifestations of TBD include a vast range of clinical findings (107). These include interstitial lung disease, particularly pulmonary fibrosis, and liver cirrhosis, often diagnosed and managed by various subspecialists. We inquire about hearing loss of any type and, if clinically indicated, pursue testing.

### Examining the Family History: A Cautionary Tale of Phenotype Variability and Non-Hematopoietic Manifestations of Germline Predisposition

Family history provides important clues to an underlying germline predisposition; however, a negative family history does not rule out a genetic disorder. Attention should be paid to the hematologic and non-hematologic manifestations of germline predisposition conditions. Given the variable expressivity and penetrance of many of these conditions, with limited genotype–phenotype correlations described to date, clinical manifestations may vary or even be absent between different family members carrying the same genetic predisposition mutation. For example, studies in kindreds with *GATA2* deficiency have reported that by age 40 years, 80% of affected individuals presented with either hematologic malignancies or infections (9, 103). However, the prevalence of clinical manifestations of germline predisposition should be interpreted with caution given potential confounding factors such as selection and ascertainment bias, and mechanisms of somatic genetic reversion discussed later.

A negative family history of MDS or other hematologic malignancies should not preclude consideration of an underlying germline genetic etiology of MDS. Silent clinical presentations have been described in a number of germline MDS syndromes. Disease manifestation may present at various ages within a kindred with the same germline genetic mutation (112). Disease anticipation has been described for some of these conditions, with clinical features being more severe and earlier in onset for each subsequent generation (113). Finally, the germline syndrome may be due to a *de novo* or recessive mutation. **Table 1** lists many clinical features variably associated with germline predisposition, which can be used as a general guide; however, absence of these findings does not rule out a germline condition. As the inheritance of these conditions may be autosomal dominant or recessive, or X-linked (**Table 1**), in addition to the history of immediate family members (parents, grandparents, children, and siblings), querying the history of extended family members may also be illuminating.

It is important to inquire about family members with any cytopenias, need for transfusions, growth factor support, or splenectomy as possible therapy for thrombocytopenia. If there is a history of antecedent cytopenia, the type, duration, and severity of the cytopenia, prior diagnostic evaluation (such as bone marrow results), and responses to any prior treatments should be queried. Inquiring about clinical symptoms of bleeding, frequent infections, and chronic anemia will address any cellular dysfunction. For example, bleeding symptoms disproportionately severe relative to the platelet counts may be a clue to *RUNX1-*FPD or *ANKRD26* mutations. A family history of recurrent infections of any type, but particularly viral (HPV caused warts in *GATA2* deficiency), opportunistic infections [herpes zoster reactivation, primary CMV with retinitis/encephalitis, *Pneumocystis jirovecii* pneumonia, and *Candida* esophagitis in TBD (104, 107, 114)] or atypical bacterial (mycobacterial infections in *GATA2* deficiency), may suggest the presence of concurrent immunodeficiency and should prompt a more careful laboratory evaluation.

We inquire about any family members with previous diagnoses of hematologic malignancies, such as MDS, myeloid or lymphoid leukemia, or lymphoma, and the age at which these diagnoses were made. A diagnosis of a malignancy at an unusually young age should be noted. Germline predisposition, such as those associated with mutations in hematopoietic transcription factors *RUNX1, ANKRD26, GATA2*, and *ETV6*, can present with different types of hematopoietic malignancies, including MDS, AML, T-cell ALL, B-cell ALL, and myeloproliferative neoplasms (MPNs). A family history of chemotherapy sensitivity or multiple malignancies also raise suspicion for a potential germline disorder. Solid organ or head and neck cancers may be presenting features of conditions such as FA, TBD, LFS, or DBA. A family history of clinical features associated with genetic predisposition should also raise suspicion.

Finally, a history of consanguinity in the family may raise the possibility of autosomal recessive syndromes. There are rare genetic associations with founder variants in germline predisposition, for example, North African *XPC* founder mutation associated with xeroderma pigmentosum subtype C (67, 68) and the *ERCC6L2* mutation in the Finnish population (98). Thus, asking about ancestral origins may guide the differential diagnoses.

### Telling Signs in the Physical Examination

The physical exam may provide additional clues to an underlying germline predisposition in the context of an MDS diagnosis; however, the lack of syndromic features (**Table 1**) does not rule out an underlying germline predisposition.

Short stature relative to predicted height is one of the most telling physical exam findings suggestive of a predisposition condition in an adult with MDS (**Table 1**). In a large study of MDS patients who underwent stem cell transplantation, among patients younger than 40 years of age, biallelic *SBDS* mutations were associated with significantly lower height percentile (2). Skin hypo- and hyperpigmentation are features of TBD and may also be present with FA, where café-au-lait spots are often observed. A careful oral exam, especially in patients with suspected TBD and FA, should be conducted. Suspicious oral lesions should be referred to an otolaryngologist for further evaluation. Patients with FA are at high risk for squamous cell carcinomas (99). Exam of extremities should note any type of thumb abnormalities found in FA (for example, hypoplasia or pouce flottant, “floating” thumb, with associated radius abnormalities), DBA (for example, small, large, or triphalangeal, finger-like, thumb), and *MECOM-*associated syndrome (radioulnar synostosis). Hand and forearm X-rays may be helpful to diagnose findings not readily apparent on exam if clinical suspicion is high.

### Laboratory Evaluation

In addition to the complete blood count with differential and reticulocyte count at presentation, prior blood count reports are helpful to detect pre-existing cytopenias suggestive of a pre-existing disorder. Liver function tests are helpful in consideration of TBD and FA. To screen for immunological abnormalities, we typically test lymphocytes subsets for absolute counts of CD3^+^ T-cells, with CD4 and CD8 subpopulations, CD19^+^ or CD20^+^ B-cells, and CD16^+^ or CD56^+^ NK cells. We also screen for levels of IgG, IgA, and IgM immunoglobulins. Combined cytopenias and lymphocytopenias or lymphocyte dysfunction (low IgM or IgG levels) may flag a potential germline condition. B- or NK cell lymphocytopenias may be present in *GATA2* deficiency, and abnormal Ig levels may be present in *GATA2* deficiency and TBD. It should be noted that lympocytopenia (low absolute lymphocyte count, ALC, of <1.2 × 10^9^/L) has been described in MDS (115, 116). Here, again, a comparison of the ALC with previous values, if available, could be helpful to distinguish pre-existing abnormalities from those associated with MDS.

Chromosomal breakage testing for FA should be interpreted with caution in patients with MDS, particularly if peripheral blasts are present or if prior cytoreductive therapy has been administered. FA chromosomal breakage test is typically performed on peripheral blood leukocytes exposed to clastogenic agents diepoxybutane (DEB) and mitomycin C (MMC). Increased chromosomal breaks and fusions with radial forms are observed in FA. FA chromosomal breakage testing in the blood may be non-diagnostic due to peripheral blood somatic mosaicism resulting in two distinct populations of cells, where one of the populations harbors the germline mutation, while the other has a somatic genetic rescue that corrects the hypersensitivity to DNA cross-linking agents (47, 48). Skin fibroblasts can help diagnose blood mosaicism. The FA chromosomal breakage tests can be affected by prior chemotherapy or radiation therapy, since the lymphocytes may manifest baseline chromosomal breakage, however, a further increase in chromosomal breakage with the addition of DEB or MMC would support the diagnosis of FA. In such cases, performing FA testing on skin fibroblasts may be helpful. The results of the FA chromosome breakage test should be interpreted with caution and with advice from experts in the field. The diagnosis of FA informs the therapeutic plan, particularly for stem cell transplantation conditioning regimen given the sensitivity to genotoxic agents, making this is a critical part of MDS evaluation.

The telomere length testing for TBD is available clinically using a flow-FISH assay (117); however, interpretation can be challenging in the context of MDS, particularly following prior cytoreductive therapy, which might result in secondary telomere shortening. In untreated MDS patients without excess blasts, telomere lengths less than the first percentile for age in at least three different lymphocyte subsets are highly suspicious for a primary disorder of telomere maintenance (118, 119). It should be stressed that this parameter, although highly predictive in children, is not as well defined in adults, where the telomere length may fall above the first percentile for age even with known underlying genetic mutations related to telomere biology (11). If telomere measurement is available only in a single lymphocyte subset and is less than the first percentile for age, this could be suggestive of an underlying TBD and should be correlated with personal or family history of clinical features of TBD and/or germline genetic lesions. Shortening of telomeres in the granulocytes is a non-specific finding and, in isolation, is insufficient to flag a TBD. The analysis of telomere length in patients with MDS needs further study. Flow-FISH telomere length measurement must be interpreted with caution in a patient with MDS, and the potential influence of excess blasts or other secondary hematologic stressors has not been defined for this assay. In the research setting, the telomere length in MDS measured using real-time quantitative polymerase chain reaction (qPCR) analysis has been described to be shorter than in healthy individuals and is independently related to outcome in MDS after stem cell transplantation (120). The telomere length should be integrated with the clinical context, personal or family history of any TBD-related manifestations, other laboratory findings suggestive of a genetic telomere disorder, and genetic testing (see below).

Additional focused laboratory testing may be considered if clinically indicated. Evaluation of pancreatic insufficiency in SDS or SDS-like syndromes includes a pancreatic isoamylase for anyone older than 3 years old. Abdominal imaging may be helpful to assess for pancreatic lipomatosis or an atretic/abnormal pancreas suggestive of SDS. If a prior history of significant idiopathic macrocytosis or non-immune red cell aplasia is identified, the measurement of erythrocyte adenosine deaminase may be considered for evaluation of DBA.

### Bone Marrow Evaluation and Cytogenetic Assessment

One of the distinguishing features of pediatric MDS, and MDS associated with predisposition conditions, is hypocellularity of the marrow unlike adult-onset MDS, which is typically hypercellular. Up to 10%–15% of MDS in adults may be hypocellular with bone marrow cellularity of <30%, and it is not yet known if this indicates an association with a germline predisposition. Studies in adult patients have found that MDS with hypocellular marrows is observed in younger patients and is associated with severe cytopenias, when compared to MDS with normocellular or hypercellular marrows (21, 121).

Some germline predisposition conditions are associated with baseline dysplasia (122), which may be confused with MDS. Megakaryocyte dysmorphology is a frequent baseline finding in the absence of MDS in *GATA2* deficiency (123), *RUNX1-*FPD (124), or other germline predisposition conditions (125, 126). Megakaryocyte immunostaining in the bone marrow with CD61 helps highlight atypical megakaryocytes, which may be difficult to identify with hematoxylin and eosin (H&E) stain. Baseline myeloid dysplasia with nuclear hypolobation (Pelger–Huët-like cells) and hypogranulation is often seen without MDS in patients with SDS (127). Baseline mild dysmorphology is also observed in FA, including megaloblastic erythroid precursors, Pelger–Huët-like neutrophils, and hypolobated megakaryocytes, and should be considered in the diagnostic pathology (126). In such cases, serial marrow exams showing progressively increasing dysplasia, high-risk cytogenetic findings (for example, monosomy 7 or deletion of 7q, complex cytogenetic abnormalities), certain patterns of high risk somatic clones [for example, rising mutant *TP53* clone size in SDS (96)], or increased blasts would raise concern for MDS.

We routinely perform cytogenetic evaluation and FISH staining on all MDS marrows. Since the proportion of abnormal cells may be small particularly if the marrow is hypocellular, and/or these cells may have a relative disadvantage of growth and missed in cytogenetic testing, FISH testing may be helpful irrespective of cytogenetic findings (128). Complete or partial loss of chromosome 7 is the most common cytogenetic abnormality in pediatric MDS and is the most common cytogenetic abnormality observed in MDS associated with predisposition conditions (129, 130). Monosomy 7/deletion of 7q has been found in up to one-third of MDS and AML associated with FA, SDS, *GATA2* deficiency, and SCN, and has been described in MDS associated with *RUNX1-*FPD, TBD, and *ERCC6L2 (*129). In a cohort of adolescent patients with monosomy 7 MDS, the prevalence of germline *GATA2* deficiency was reported to be 72% (6). Isochromosome 7q [i(7)(q10)] or del(20q) are cytogenetic abnormalities found in SDS, but these clones have not been associated with progression to malignancy (91, 131). Other common cytogenetic abnormalities described with germline predisposition conditions include trisomy 8 with *GATA2* deficiency (6) and gains of chromosomes 1q and 3q with FA (126).

### Genetic Testing for Germline Predisposition

The availability of clinical next-generation sequencing (NGS) has revealed the complex landscape of germline mutations in myeloid neoplasms, including MDS. Many germline mutations underlying predisposition conditions are also often found as somatic mutations in myeloid leukemias and may first be picked up on a somatic mutation panel as part of the diagnostic evaluation for MDS. Distinguishing germline from somatic mutations, particularly in consideration of germline predisposition conditions in young patients, has added to the complexity of interpretating the NGS result and carries profound clinical implications discussed below.

Most genetic panels designed for somatic mutation analysis of myeloid malignancies target mutations recurrently found in adult hematologic malignancies. Commercial somatic and germline NGS panels differ with respect to what genes and gene regions are included. Analytic pipelines for germline *versus* somatic mutations are also distinct. Additionally, many of these somatic genetic panels can miss deletions or duplications in a gene (for example, in *GATA2* deficiency and *RUNX1-*FPD) and changes in non-coding regions like promoter regions (*ANKRD26*) and introns (*GATA2*) that may underlie germline predisposition conditions. Indeed, a normal/negative somatic NGS panel does not rule out a germline genetic cause of an MDS diagnosis. In our practice, we utilize a somatic NGS panel of 88 genes to evaluate somatic MDS mutations (132), accompanied by a separate gene panel designed for genetic analysis of germline variants. The identification of variants in genes known to be mutated either somatically or in the germline, such as *RUNX1*, *GATA2*, and *TP53*, warrant further consideration regardless of variant allele frequency, which may be affected by confounding factors such as copy number variants, loss of heterozygosity, or somatic reversion. Approaches to distinguish germline from somatic mutations include examination of non-hematopoietic tissues such as skin fibroblasts and persistence of the variant at a constant appropriate variant allele fraction (VAF) in remission marrows after MDS-directed therapy. For example, we reported a case of an adolescent patient with *RUNX1*-FPD who presented with B-ALL, who had a personal and family history of mild bleeding symptoms. She was found with a *RUNX1* variant on the NGS panel with a VAF of 52%, initially classified as a variant of unknown clinical significance (VUS). Remission marrow showed persistence of this variant at a similar VAF, which was confirmed to be a germline with fibroblast sequencing and positive testing of her father who had long-standing bleeding symptoms (133).

The designation of a VUS indicates lack of sufficient evidence to meet current American College of Medical Genetics (ACMG) requirements for pathogenic/likely pathogenic or benign/likely benign designation. Many germline variants classified as VUS have not been described in the literature or in publicly available databases gene variants such as ClinVar (https://www.ncbi.nlm.nih.gov/clinvar) or gnomAD (https://gnomad.broadinstitute.org). We work closely with experts in specific genes or diseases and colleagues in genetic counseling to analyze these variants with the clinical history, laboratory evaluation, association of the variant within family pedigree phenotypes, variant allele frequencies in control populations, and gene-specific structural and functional data (134). There are ongoing research and collaborative expert panel recommendations such as Clinical Genome Consortium (ClinGen: https://clinicalgenome.org/) for the interpretation and classification of germline variants (135). For specific gene/disease pairs, such as for *RUNX1, TP53*, and others, guidelines are curated by a Myeloid Malignancy Variant Curation Expert Panel (MM-VCEP) and include additional functional assessment guidelines to enhance the accuracy of variant interpretation (136–138). Variant classification is a highly active area of research, and consultation with an expert in the field can be helpful.

Interpretation of the sequencing results may be confounded by somatic genetic rescue (SGR) (139). In a number of these syndromes, SGR results in the correction of the germline mutation in a single hematopoietic stem and progenitor cell, which then results in a selective growth of the wild-type corrected clone over time, with reduction in the relative presence of the germline mutant cells in the hematopoietic tissues (97). Examples of SGR or reversion reported in literature are listed in **Table 1**. As such, VAFs of true germline mutations will be lower than the expected heterozygous allele frequencies, which may result in the omission of the variant by the analytic pipeline for germline mutations or mis-attribution to a somatic origin. We have limited understanding whether the remaining abnormal germline clones have the same high risk of malignant transformation. For example, in FA, revertant mosaicism in the blood may improve overall blood counts (47, 140), but still confers a risk for leukemogenesis (141), while in the rare single cases of reversion in *GATA2* deficiency (64) and *RUNX1-*FPD *(*66), hematologic malignancies have not been reported. A curious example of SGR in hematopoiesis is the case of *SAMD9* and *SAMD9L* syndromes. *SAMD9* and *SAMD9L* are located near each other on chromosome 7q21.3. Gain-of-function (GOF) mutations in these genes are associated with childhood cytopenias and high risk of developing monosomy 7 or 7q− MDS (142). GOF mutations in stem and progenitor cells result in reduced proliferation, and thus, a cellular competitive advantage is gained by a clone that losses the mutant allele. SGR in patients with germline SAMDL/SAMD9L mutations may occur through several mechanisms: gene correction *via* genetic reversion or copy neutral loss of heterozygosity (uniparental disomy), inactivation of the mutant allele by a somatic loss of function mutation within the mutant allele, or through complete or partial loss of the chromosome harboring the mutant allele (chromosome 7), termed adaptation by aneuploidy (69, 72, 143, 144). The last mechanism generates a monosomy 7 or deletion of 7q clone(s), which may progress to myeloid malignancy. The germline mutations may be detected by sequencing a non-hematopoietic tissue such as a skin fibroblast. Some centers routinely test skin fibroblasts for the evaluation of germline genetic predisposition in patients with MDS.

### Altering MDS Care for Patients With Germline Predisposition

The recognition of the underlying germline predisposition informs the approach to therapy in MDS. A therapy with curative intent will often involve stem cell transplantation (SCT) where donor selection is a critical part of the workup. Identification of a germline genetic predisposition allows evaluation of potential family member donors to avoid choosing an affected but clinically asymptomatic donor (145). If a pathogenic germline mutation is identified in the proband, targeted sequencing of the suspected germline variant should be evaluated in the potential family donor. As genetic testing of the donor is time sensitive, the sequencing platform that can most rapidly return test results should be utilized. If the workup of the patient reveals abnormal functional test results, for example, short telomeres concerning TBD, without identification of a specific gene mutation, then evaluation of the related donor for that functional test and a bone marrow examination to rule out any significant hematologic abnormalities should be considered. In adult patients with MDS, the diagnosis of a germline MDS predisposition will also inform the decision around SCT *versus* chemotherapy or other alternative therapeutic measures, as SCT will not only cure the disease at hand but also eliminate the future heightened risk of hematologic malignancy (146).

Beyond donor selection, identification of germline predisposition conditions may change the conditioning regimen used in SCT to reduce non-relapse mortality (NRM). NRM is a significant complication in SCT for many predisposition conditions such as TBD (147) and FA, where there is a clear benefit for reduced intensity conditioning (RIC). For pediatric patients with MDS, SCT often involves myeloablative conditioning (MAC) regimens, although some RIC regimens are also used. A randomized trial in adults with MDS showed comparable relapse outcomes for patients receiving RIC *versus* MAC (148). Hence, the presence of certain germline predisposition conditions, particularly those associated with DNA-damage repair mechanism or high NRM, may benefit from receiving RIC and not MAC regimens to reduce NRM. Lastly, SCT outcomes may be affected by the underlying germline condition and associated comorbidities (2, 120) and should be considered at the time of transplantation planning.

Caring of patients during and after SCT for MDS may also be influenced by a germline predisposition condition. For example, in a single center cohort of patients undergoing SCT for *GATA2* deficiency-related blood disorders, we found an increased risk of thrombotic and neurological complications, compared to patients with *GATA2* wild-type MDS or ALL (147). A second cohort of patients with *GATA2* deficiency-related MDS who underwent SCT reported four cases of neurological complications and several patients with transplant-associated thrombotic microangiopathy (149). Patients may also experience clinical signs of non-hematologic organ toxicity, which do not correct with SCT, such as *GATA2* deficiency-related lymphatic dysfunction, or lung and liver disease in TBD. Attention to post-SCT monitoring is needed for a patient with germline predisposition conditions with an increased risk of solid tumors [for example, FA, LFS, *GATA2* deficiency (103), and DBA (150)].

### Considerations for Family Counseling

Diagnosis of germline predisposition conditions informs family counseling. In the current era of successful oncofertility programs, young patients undergoing MDS-directed therapy, including SCT, have an opportunity to consider family planning options after their treatment. At the appropriate time, genetic counselors are helpful to engage in discussions about prenatal counseling, preimplantation genetic testing, and other possibilities.

Testing of family members should be discussed to allow informed decision-making and to guide their health maintenance. Testing of siblings, particularly those who may be considered for stem cell donation, is often a first step in this process. Testing of all direct family members (parents, siblings, and children) of the affected patient and interested biologically related members of the extended family should be discussed together with a genetic counselor with expertise in these conditions.

## Concluding Remarks

We have gained valuable insight about MDS pathogenesis from pediatric MDS with germline predisposition conditions. Expanding the systematic evaluation for these conditions to adult patients with MDS will guide medical management and improve outcomes with tailored therapies, appropriate management of co-morbidities, and inform donor selection. Hematologists should be alert to potential red flags from personal and family history, physical exam, blood and marrow examination, and somatic NGS results, which are suggestive of genetic predisposition in adult patients with MDS.

Investigations into germline predisposition to hematologic malignancies continue to address unanswered questions of disease penetrance, incidence, mechanisms of somatic genetic rescue, and prognostic markers of MDS risk once a conditions is identified in asymptomatic patients. Adults with germline predisposition to MDS may present without a suggestive childhood history. The identification of older adult-onset MDS for genetic predisposition conditions such as *DDX41* or TBD-related MDS has expanded the age spectrum of consideration of germline conditions in MDS. Increasing awareness and evaluation for genetic predisposition to MDS in the diagnostic workup of adult patients will improve patient care, guide therapies, and inform monitoring of toxicities and comorbidities with the goal of improving patient outcomes.

## Author Contributions

All authors listed have made a substantial, direct, and intellectual contribution to the work and approved it for publication.

## Funding

This work was supported by NIH/NIDDK 1RC2DK 122533 to AS.

## Conflict of Interest

The authors declare that the research was conducted in the absence of any commercial or financial relationships that could be construed as a potential conflict of interest.

## Publisher’s Note

All claims expressed in this article are solely those of the authors and do not necessarily represent those of their affiliated organizations, or those of the publisher, the editors and the reviewers. Any product that may be evaluated in this article, or claim that may be made by its manufacturer, is not guaranteed or endorsed by the publisher.
